# Evaluating the predictive performance of malaria antibodies and *FCGR3B* gene polymorphisms on *Plasmodium falciparum* infection outcome: a prospective cohort study

**DOI:** 10.1186/s12936-020-03381-8

**Published:** 2020-08-27

**Authors:** Duah Dwomoh, Bright Adu, Daniel Dodoo, Michael Theisen, Samuel Iddi, Thomas A. Gerds

**Affiliations:** 1 Department of Biostatistics, School of Public Health, University of Ghana, Accra, Ghana; 2 Department of Immunology, Noguchi Memorial Institute of Medical Research, College of Health Sciences, University of Ghana, Accra, Ghana; 3 Department for Congenital Disorders, Statens Serum Institut, Copenhagen, Denmark; 4 Centre for Medical Parasitology at Department of International Health, Immunology and Microbiology, University of Copenhagen, Copenhagen, Denmark; 5 Department of Infectious Diseases, Copenhagen University Hospital, Rigshospitalet, Copenhagen, Denmark; 6 Department of Statistics and Actuarial Sciences, University of Ghana, Accra, Ghana; 7 Section of Biostatistics, Department of Public Health, University of Copenhagen, Copenhagen, Denmark

**Keywords:** Apical membrane antigen 1, *FCGR3B* gene polymorphisms, Area under the receiver operating characteristic curve, Brier score, Bootstrap-validation, Calibration, Discrimination, Malaria, Antibodies, Antigenes

## Abstract

**Background:**

Malaria antigen-specific antibodies and polymorphisms in host receptors involved in antibody functionality have been associated with different outcomes of *Plasmodium falciparum* infections. Thus, to identify key prospective malaria antigens for vaccine development, there is the need to evaluate the associations between malaria antibodies and antibody dependent host factors with more rigorous statistical methods. In this study, different statistical models were used to evaluate the predictive performance of malaria-specific antibodies and host gene polymorphisms on *P. falciparum* infection in a longitudinal cohort study involving Ghanaian children.

**Methods:**

Models with different functional forms were built using known predictors (age, sickle cell status, blood group status, parasite density, and mosquito bed net use) and malaria antigen-specific immunoglobulin (Ig) G and IgG subclasses and *FCGR3B* polymorphisms shown to mediate antibody-dependent cellular functions. Malaria antigens studied were Merozoite surface proteins (MSP-1 and MSP-3), Glutamate Rich Protein (GLURP)-R0, R2, and the Apical Membrane Antigen (AMA-1). The models were evaluated through visualization and assessment of differences between the Area Under the Receiver Operating Characteristic Curve and Brier Score estimated by suitable internal cross-validation designs.

**Results:**

This study found that the *FCGR3B*-c.233C>A genotype and IgG against AMA1 were relatively better compared to the other antibodies and *FCGR3B* genotypes studied in classifying or predicting malaria risk among children.

**Conclusions:**

The data supports the *P. falciparum*, AMA1 as an important malaria vaccine antigen, while *FCGR3B*-c.233C>A under the additive and dominant models of inheritance could be an important modifier of the effect of malaria protective antibodies.

## Background

Malaria remains a major public health concern globally and is considered as one of the most prevalent and lethal human infectious diseases among children in sub-Saharan Africa [1]. Despite the drastic reduction in the number of malaria cases and deaths in all ages globally, it still accounts for 10% of child deaths in sub-Saharan Africa [1], and mortality is mostly higher among children below the age of 5 years. Individuals in endemic regions increasingly develop immunity to malaria with age and this is conventionally thought to reflect a slow and gradual acquisition of protective antibodies [2]. Asymptomatic carriers may be a reservoir for malaria transmission [3]. It has recently been shown that interaction between naturally acquired antibodies to *Plasmodium falciparum* and polymorphisms in host *FCGR3B* gene, encoding the Fc Gamma Receptor IIIB (FcγRIIIB) plays a key role in immunity against malaria [4]. The FcγRIIIB is exclusively expressed on human neutrophils and crosslinking with immunoglobulin (Ig) G antibodies mediates neutrophil degranulation and generation of reactive oxygen species (ROS) [5], which kills intra-erythrocytic *P. falciparum* [6]. It is conceivable that other host genes may also modify the protective effect of malaria antibodies. This emphasizes the need for robust modelling approaches to effectively address such confounders in malaria vaccine studies. It is quite plausible that the long delay in attaining an effective malaria vaccine may partly be due to inadequacies of traditional statistical approaches used in malaria immuno-epidemiological studies to determine the performance of predictors in classifying or predicting malaria risk [7–10]. Traditionally, most studies use generalized linear models (GLM) depending on the measurement scale of clinical malaria. GLM provides an extensive class of tools for modelling the effect of predictors. Statistical prognostic modelling techniques have been applied primarily in the area of non-communicable diseases such as cardiovascular diseases and lung cancer. For instance, Gail et al. [11] developed a model of breast cancer risk prediction and implications for chemoprevention which was later validated by Rockhill et al. [12]. Several risk prediction models for other cancers and cardiovascular diseases [12–21] have also been developed. For clinical malaria, on the other hand, personalized risk estimation has not been extensively studied. As indicated by several authors [7–10, 22], markers such as polymorphisms and antigen-specific antibodies proposed for classifying or predicting risk in individual subjects must be held to a much higher standard than just assessing associations based on odds ratio estimates. Pepe et al. [22] showed that strong statistical associations (including odds ratio, relative risk) between disease and host-specific factors found in literature do not necessarily imply that those factors can discriminate between a subject who is likely or not have the disease in a specified time. A risk prediction model exploits the joint predictive power of several variables on the risk of an event or disease. A robust malaria risk prediction model based on epidemiological predictors may contribute to finding possible answers to the question of which parasite antigens and host factors should be the main research focus in the efforts to find optimal control strategies and vaccines. This is particularly important as the number of malaria-specific antibodies and host gene polymorphisms found to be associated with clinical malaria have increased significantly over the past few years [3] but with little impact on malaria control. Using a more rigorous prediction modelling approach, this study aims to evaluate the predictive performance of malaria antibodies and *FCGR3B* gene polymorphisms on *P. falciparum* infection outcome. To assess the performance of the models, this study used Brier scores and area under the receiver operating characteristic curve (AUROC) through appropriate bootstrap cross-validation design.

The identified model was obtained by comparing Brier score estimates and the AUROC curve of several models that integrate malaria antibodies and host gene polymorphisms.

## Methods

### Data source

Data used for modelling the risk of malaria was secondary data obtained from a prospective longitudinal malaria cohort study which was conducted from May 2008 to January 2009 among children under 13 years of age in five different communities in the Shai Osudoku (formerly Dangme West) district of Ghana (Asutsuare, Kewum, Mafikorpe, Osuwem, and Volivo) [20, 21]. These children were observed both actively and passively for malaria case detection. The primary outcome measure was clinical malaria defined as fever with any level of *P. falciparum* parasitaemia plus at least one clinical symptom of malaria, such as vomiting, joint pains, diarrhoea. In this study, the term “Susceptible” and “Protected” are used to represent clinical malaria and no malaria case detection respectively over the study period. The proportion of children that developed malaria in the 1 year follow up was 15.0% (incidence proportion) and there were approximately 1.7 malaria cases per 100 children per month (incidence rate) [23]. The predictors of clinical malaria included age in years, sex, sickle cell status, blood group, haemoglobin level, malaria antigen-specific immunoglobulin (Ig) G and subclasses (IgG1, IgG2, IgG3, and IgG4) and *FCGR3B* polymorphisms (c.108C>G-rs403016, c.114T>C-rs447536, c.194A>G-rs448740, c.233C>A-rs5030738, c.244A>G-rs428888 and c.316A>G-rs2290834). Malaria antigens studied were Merozoite surface proteins (MSP-1 and MSP-3), Glutamate Rich Protein (GLURP)-R0, R2, and the Apical Membrane Antigen (AMA-1). Antibody levels were measured by sandwich ELISA and optical density values converted to antibody units using a four parameter curve fitting program by means of a reference curve on each plate generated by serial dilution of malaria hyperimmune sera [3].

This study includes a summary of the climatic variables at the time of the study to serve as a guide for future studies that may wish to compare their findings to this study. Changes in relative humidity (RH), which is the ratio of the partial pressure of water vapor relative to saturated vapour at the specified temperature was assessed over the study period. The minimum and maximum relative humidity over the study period were 71.0% and 98.0% respectively. The total monthly rainfall ranged between 0 and 273.5 mm with the month of May 2008 recording the highest total rainfall. The total rainfall between June and December 2008 ranged from 19.7 to 125.4 mm. The maximum daily rainfall was recorded in the month of June 2008 (60 mm). There was no rainfall in the month of January 2009. The minimum and maximum air temperature ranged from 19.3 to 36.8 °C.

### Malaria risk prediction model and performance measures

Let $D_{n} = \left\{ {Y_{i} ,\varvec{X}_{i} } \right\}_{i = 1, \ldots ,n}$ be a malaria data set with *n* number of children aged less than 13 years. $\varvec{X}_{i} = \left( {X_{ip} } \right)_{p = 1, \ldots ,P, i = 1, \ldots ,n}$ be an input matrix of *P* predictors of malaria (age, antibodies) and

$$ Y_{i} = \left\{ {\begin{array}{*{20}c} {1\;if\;the\;child\;is\;positive } \\ {0\;if\;the\; child\; is\; negative} \\ \end{array} } \right. $$

Let $G \subseteq \left\{ {1, \ldots G} \right\}$ be a subset of the available predictors where $p = p_{1} ,p_{2} , \ldots ,p_{g}$. Let $\beta = \beta_{1} ,  \beta_{2} , \ldots ,\beta_{g}$ be the regression coefficient to be estimated. The predicted risk of malaria is modelled using the logistic regression model. Specifically, for the trained prediction model, $\hat{\tau }_{n}$ which assigns to each child the probability of developing clinical malaria, the estimated malaria risk prediction model is given by:

$$ P\left( {Y_{i} = 1|\varvec{X}_{i} } \right) = \hat{\tau }_{n} \left( {\varvec{X}_{i} } \right) = \hat{\tau }_{n} = \left[ {1 + \exp \left\{ {\hat{\beta }_{0} - \mathop \sum \limits_{g \in G} \hat{\beta }_{g} \left( {X_{p} } \right)} \right\}} \right]^{ - 1} $$

where $\hat{\beta }$ is a vector of estimated parameters of the model associated with predictor variables, $\hat{\beta }_{0}$ is the estimated intercept of the logistic regression model considered as the baseline risk of malaria and $\hat{\tau }_{n} \left( {\varvec{X}_{i} } \right)$ is the predicted risk of malaria for the *i*th child with baseline characteristics $\varvec{X}_{i}$.

The modelling strategy was to first identify a baseline model out of several other competing models relating to the prevalence of malaria to baseline covariates. Comprehensive discrimination and calibration assessments of the fitted models were explored based on the AUROC curve, Brier score, root mean squared error and the total explained variations in predicted probabilities (*R*^2^) using bootstrap cross-validation from 200 bootstrap samples with replacement. In selecting predictor variables to be included in the final model, candidate predictor variables were screened using tools for discriminative and calibration abilities such as the area under the receiver operating characteristics curve, the Brier score and explained variation in predicted risk. The model’s ability to predict accurately was further assessed by means of the calibration plot. According to Gerds et al. [24], the values of the Brier score could be interpreted as the loss or regret which is incurred when the prediction model $\hat{\tau }_{n}$ is applied to a child whose true malaria status is $Y_{i} .$ Model performance was assessed via bootstrap cross-validation. Selection of predictor variables (age, sickle cell, blood group, haemoglobin, parasite density and, mosquito net use) for the baseline model was premised on subject matter knowledge and prior evidence of association with the risk of malaria from literature. Continuous predictors were fitted via restricted cubic splines. To quantify over-fitting and to recalibrate the model, the heuristic shrinkage estimator $\hat{\gamma } = \frac{{model\chi^{2} - p}}{{model\chi^{2} }}$ was used where *p* is the number of predictors (regression parameters including both linear and non-linear and possible interaction terms), $\chi^{2}$ is the likelihood ratio $\chi^{2}$ test statistic computed using the full set of *p* parameters to determine whether any of the predictor(s) is/are associated with log-odds of developing clinical malaria. For the model to be calibrated well for future data, $\hat{\gamma }$ was multiplied by $X\hat{\beta }$ and that defines shrinkage. The penalty factor was determined by means of repeated cross-validation of the data. All antigen-specific antibodies were log-transformed to base *e* in subsequent analysis. All models were fitted with R programming software version 3.2.4 with the following specialized packages: Design [25], Penalized [26]. Data cleaning and all other forms of data preparations were done with Stata SE version 13. A *p*-value of < 0.05 was considered statistically significant.

## Results

### Description of study participants and clinical malaria distribution

The study recruited 799 children of which 393 (49.2%) were males and 406 (50.8%) were females. Complete information on candidate predictors was available for 395 children. The missing data were due to children for whom either no or insufficient plasma was available for all antibody measurement. Additionally, children for whom there was no DNA or poor quality DNA that resulted in poor genotyping data were excluded. The overall median age for this study sample was 5.0 years (Interquartile range = 3.0–8.0). The cumulative incidence of malaria was 13% (53 out of 395 children). The bed net use among the children was 40.5%. The analysis of sociodemographic characteristics and baseline biomarkers on the risk of clinical malaria indicated that the cumulative incidence of malaria did not differ significantly among the baseline predictors that were studied (*p*-value ≥ 0.05). Distribution of other predictors and clinical malaria status can be found in Table 1.

**Table 1:** Bivariate analysis of malaria predictors and clinical malaria

Predictor	Levels	Protected	Susceptible	Combined	p-value
		N = 342	N = 53	N = 395	
Age in years		5(3,8)	5 (3,7)	5 (3,8)	0.12^e^
Mosquito net use	No	60% (204)	58% (31)	59% (235)	0.87^f^
Blood group	A	18% (63)	19% (10)	18% (73)	0.93^f^
	AB	6% (22)	8% (4)	7% (26)	
	B	29% (98)	25% (13)	28% (111)	
	O	46% (159)	49% (26)	47% (185)	
Sickle cell status	Positive	15% (51)	19% (10)	15% (61)	0.46^f^
Parasite count(categorized)	positive	7% (25)	4% (2)	7% (27)	0.34^f^
Haemoglobin at enrollment(gram per dL)		12 (11,13)	11 (11,12)	12 (11,13)	0.11^e^
Additive c.108C>G	CC	29% (100)	26% (14)	29% (114)	0.4^f^
	CG	42% (144)	36% (19)	41% (163)	
	GG	29% (98)	38% (20)	30% (118)	
Additive c.114T>C	CC	27% (92)	26% (14)	27% (106)	0.82^f^
	CT	43% (147)	47% (25)	44% (172)	
	TT	30% (103)	26% (14)	30% (117)	
Additive c.233C>A	AA	9% (31)	2% (1)	8% (32)	0:2^f^
	AC	27% (93)	28% (15)	27% (108)	
	CC	64% (218)	70% (37)	65% (255)	
Additive c.244A>G	GG	31% (106)	32% (17)	31% (123)	0.36^f^
	AG	39% (134)	47% (25)	40% (159)	
	AA	30% (102)	21% (11)	29%(113)	
Additive c.316A>G	GG	13% (43)	17% (9)	13% (52)	0.65^f^
	AG	32% (109)	32% (17)	32% (126)	
	AA	56% (190)	51% (27)	55% (217)	
Additive c.194A>G	GG	39% (135)	30% (16)	38% (151)	0.43^f^
	AG	39% (135)	45% (24)	40% (159)	
	AA	21% (72)	25% (13)	22% (85)	
Dominant c.108C>G	CC vs CG-GG	71% (244)	62% (33)	70%(277)	0:18^f^
Dominant c.114T>C	TT vs CT-CC	73% (250)	74% (39)	73% (289)	0:94^f^
Dominant c.194A>G	AA vs AG-GG	61% (207)	70% (37)	62% (244)	0:2^f^
Dominant c.233C>A	CC vs AC-AA	91% (311)	98% (52)	92% (363)	0:075^f^
Dominant c.244A>G	AA vs AG-GG	69% (236)	68% (36)	69% (272)	0:87^f^
Dominant c.316A>G	AA vs AG-GG	87% (299)	83% (44)	87% (343)	0:38^f^
Recessive c.108C>G	CC-CG vs GG	29%(100)	26% (14)	29% (114)	0:67^f^
Recessive c.114T>C	TT-CT vs CC	30% (103)	26% (14)	30% (117)	0:58^f^
Recessive c.194A>G	AA-AG vs GG	21% (72)	25% (13)	22% (85)	0:57^f^
Recessive c.233C>A	CC-AC vs AA	64% (218)	70% (37)	65% (255)	0:39^f^
Recessive c.244A>G	AA-AG vs GG	30% (102)	21% (11)	29% (113)	0:17^f^
Recessive c.316A>G	AA-AG vs GG	56% (190)	51% (27)	55% (217)	0:53^f^
log.IgG-MSP1		2.2 (1.6,3.7)	2.4 (1.6,3.2)	2.3 (1.6,3.6)	0:77^e^
log.IgG1-MSP1		3.2 (2.4,5.4)	3.1 (2.6,4.4)	3.2 (2.4,5.4)	0:6^e^
log.IgG2-MSP1		1.9 (1.6,2.8)	2.0 (1.6,2.7)	1.9 (1.6,2.8)	0:71^e^
log.IgG3-MSP1		3.3 (1.9,6.0)	3.1 (2.0,6.0)	3.3 (1.9,6.0)	0:83^e^
log.IgG4-MSP1		1.6 (1.4,2.0)	1.6 (1.3,1.9)	1.6 (1.4,2.0)	0:64^e^
log.IgG-MSP3		3.2 (2.5,4.6)	3.2 (2.5,4.8)	3.2 (2.5,4.6)	0:66^e^
log.IgG-1MSP3		3.2 (2.6,4.7)	2.9 (2.6,4.2)	3.2 (2.6,4.7)	0:36^e^
log.IgG-2MSP3		1.8 (1.6,2.2)	1.8 (1.5,2.1)	1.8 (1.6,2.2)	0:52^e^
log.IgG-3MSP3		2.7 (1.8,4.5)	2.6 (2.0,4.1)	2.7 (1.8,4.5)	0:89^e^
log.IgG-4MSP3		1.7 (1.4,2.1)	1.7 (1.5,2.1)	1.7(1.4,2.1)	0:82^e^
log.IgG-GLURPR0		3.4 (2.4,4.7)	3.8 (2.6,4.8)	3.4 (2.4,4.7)	0:36^e^
log.IgG1-GLURPR0		3.4 (2.5,4.9)	3.7 (2.7,5.1)	3.4 (2.5,4.9)	0:40^e^
log.IgG2-GLURPR0		1.9(1.6,2.3)	1.8(1.6,2.9)	1.9 (1.6,2.4)	0:61^e^
log.IgG3-GLURPR0		2.1 (1.6,3.4)	2.0 (1.7,2.7)	2.1 (1.6,3.4)	0:78^e^
log.IgG4-GLURPR0		1.5 (1.3,1.7)	1.5 (1.2,1.7)	1.5 (1.3,1.7)	0:44^e^
log.IgG-GLURPR2		3.9 (2.2,5.9)	4.2 (2.8,6.0)	4.0 (2.2,5.9)	0:26^e^
log.IgG1-GLURPR2		5.8 (3.8,8.0)	5.8 (4.3,7.5)	5.8 (3.9,8.0)	0:93^e^
log.IgG2-GLURPR2		3.2 (2.1,6.3)	2.9 (2.1,6.4)	3.1 (2.1,6.3)	0:56^e^
log.IgG3-GLURPR2		5.9 (3.5,7.8)	6.1 (4.1,7.3)	5.9 (3.5,7.6)	0:96^e^
log.IgG4-GLURPR2		2.1 (1.8,3.1)	2.1 (1.8,2.4)	2.1 (1.8,2.9)	0:62^e^
log.igG-AMA1		6.2 (3.6,9.2)	6.4 (4.5,8.4)	6.7 (3.8,9.1)	0:45^e^
log.IgG1-AMA1		9.5 (6.2,10.2)	8.9 (6.6,10.0)	9.4 (6.3,10.2)	0:61^e^
log.IgG2-AMA1		3.2 (2.1,4.5)	2.9 (2.1,4.2)	3.2 (2.1,4.4)	0:57^e^
log.IgG3-AMA1		4.7 (3.1,6.4)	4.8 (3.2,6.8)	4.7 (3.2,6.5)	0:54^e^
log.igG4-AMA1		4.0 (2.5,5.1)	3.6 (2.9,4.7)	3.9 (2.6,5.1)	0:62^e^

a (b, c), represent the median, lower quartile, and the upper quartile for continuous variables.$e$-Wilcoxon Ranksum test, $f$-Fishers Exact Test. Numbers after percents are frequencies. Additive model: assumes the risk associated with an allele is increased r-fold for heterozygotes and 2r-fold for homozygote; Dominant model: assumes risk association with the dominant allele and compares homozygous wild type with a combination of the heterozygous and homozygous for the variant; Recessive model: assesses risk association with the recessive allele and compares homozygous variant type with a combination of the heterozygous and homozygous for the wild type. Tests used: Wilcoxon test; Pearson test, MSP: Merozoite surface protein, GLURP: Glutamate Rich Protein, AMA: Apical membrane antigen. Note: natural log transformation was used

### Modelling results on baseline covariates and socio-demographic characteristics

**Fig. 1:**
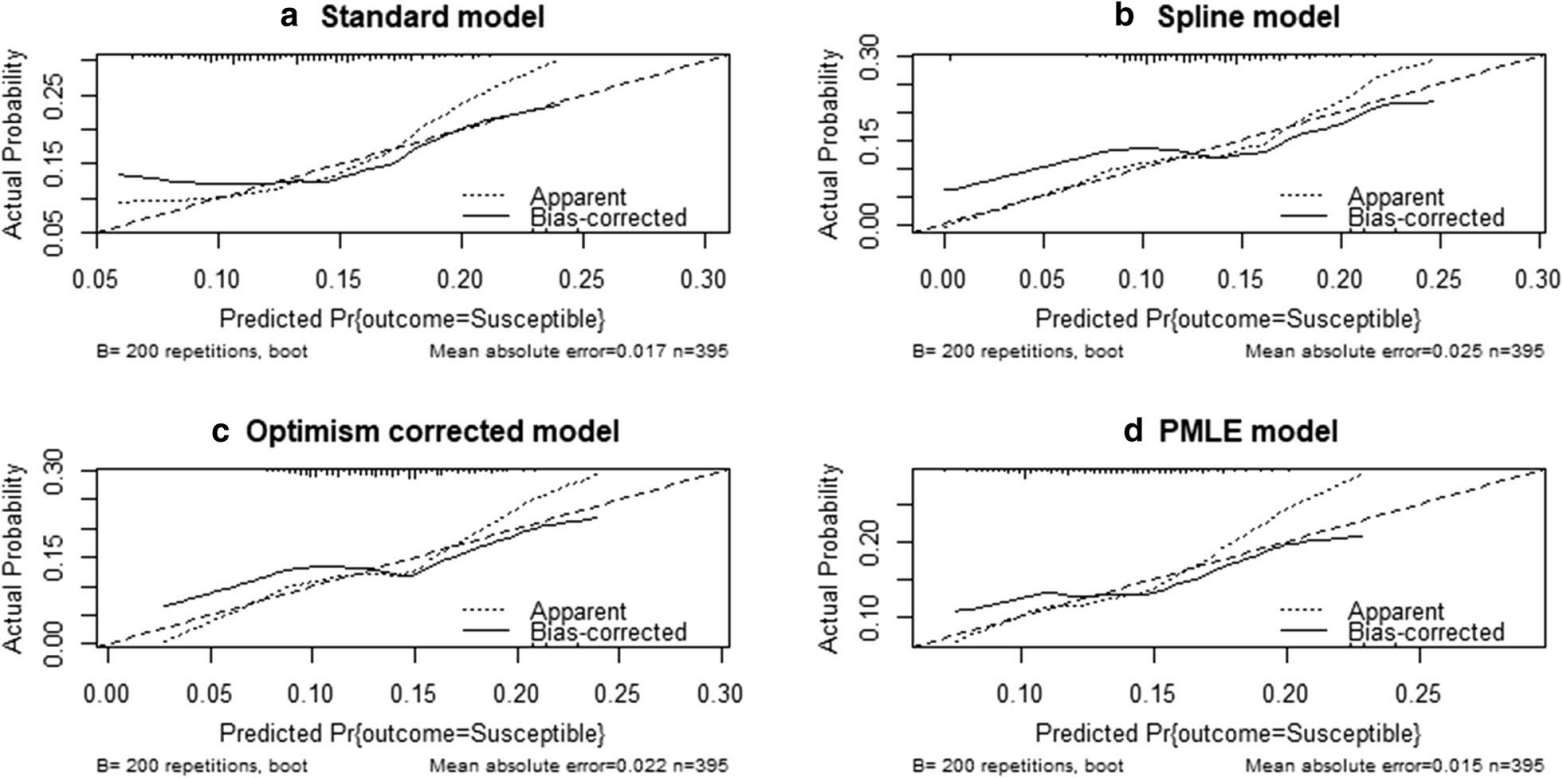
Calibration plots comparing the four baseline models

Four different initial models were rigorously assessed and the best model was chosen to serve as the baseline model using the aforementioned indices. These models are presented in Table 2. ‘Model.standard’ is a linear additive model of age in years, haemoglobin level in (g/dl), sickle cell, blood group, bed net use and parasite density categorized into positive and negative for presence and absence of parasite in blood at enrollment, respectively. The ‘Model.spline’ is the additive model (Model.standard) but it was modelled the nonlinear effect of age and haemoglobin using 5 knots restricted cubic splines, and also included an interaction term of bed net use, parasite density. The ‘Model.slope.optimism’ (SCFM) is the ‘Model.spline’ but with regression coefficients shrunk by slope corrected optimism. Finally, a ‘PMLE.model’, which is the ‘Model.spline’ but has estimates of the *β* coefficient that were based on the Penalized Maximum Likelihood Estimation procedure [27]. Upon careful consideration based on the above model prediction performance measures, the ‘PMLE.model’ was chosen as the baseline model. The bootstrap cross-validated estimates of AUROC, Brier score, root mean squared error and explained variations in predicted probabilities of the ‘PMLE.model’ are 50.0%, 10.0%, 0.32 and 17.0%, respectively. Although these performance indices were generally poor, it was better as compared to the other models (Table 2). The probability that PMLE.model will assign a higher predicted risk to a randomly chosen child with malaria compared with a randomly chosen child with no malaria is 50.0% (discrimination ability = 50.0%; Table 2), which is not better than random prediction. The Brier score of 10.0% is the expected loss or regret incurred when predicted risk from PMLE.model is issued to a child whose true malaria status is either susceptible or protected. The variations in predicted risk of malaria explained by the chosen model is 17.0% (Table 2). Furthermore, the calibration plot in Fig. 1 shows that the PMLE model underestimates children at low risk of malaria and overestimate children at higher risk of malaria.

**Table 2:** Predictive effect of socio-demographic indices and baseline covariates

Model specification	LR, p-value	BCV AUROC (%)	BCV BS(%)	RMSE	R^2^
Standard model (S)	5.34, 0.7209	51.0	12.0	0.35	0.01
Spline model	8.08, 0.8387	49.0	11.0	0.33	0.10
Slope corrected optimism model (SCFM)	7.32,0.7844	49.0	11.0	0.33	0.10
PMLE model	5.76, 0.7360	50.0	10.0	0.32	0.17

LR: Likelihood Ratio test statistic; BCV: Bootstrap cross-validation; RMSE: Root Mean Squared Error; *R*^2^: Proportion of explained variation in predicted risk; AUCROC: Area Under the Receiver Operating Characteristic curve, BS: Brier score

### Modelling results on antigen-specific antibodies and *FCGR3B* polymorphisms

The predictive effect of each antibody (IgG and subclasses) and *FCGR3B* was evaluated by introducing them one after the other in the selected baseline penalized maximum likelihood model (PMLE.model) as shown in Table 3. All antibodies were modelled via 5 knots restricted cubic spline. Admittedly, none of the antibodies nor the *FCGR3B* could significantly improve the performance of the PMLE.model after introduction but it was observed that IgGAMA1, IgG1AMA1, and *FCGR3Bc.*233*C*>*A* were better than all the other predictors in relation to their bootstrap cross-validated estimates of relatively higher AUROC, lower Brier score, and and relatively higher *R*^2^.

**Table 3:** Assessing the predictive effect of each malaria antibody and *FCGR3B* polymorphisms on the risk of malaria

Model specification	LR, p-value	BCV AUROC	BCV BS (%)	RMSE	*R*^2^
F + log.IgGAMA1	12.23, 0.0301	55	10	0.3511	2.0
F + log.IgG1AMA1	14.09, 0.0223	57	10	0.3501	2.0
F + log.IgG2AMA1	5.76, 0.8058	49	12	0.3400	1.0
F + log.IgG3AMA1	10.41, 0.7668	54	12	0.3525	2.0
F + log.IgG4AMA1	7.03, 0.7668	50	12	0.3439	3.0
F + log.IgGGLURPR0	7.41, 0.6501	51	12	0.3514	1.0
F + log.IgG1GLURPR0	6.93, 0.6987	51	12	0.3518	0.0
F + log.IgG2GLURPR0	6.38, 0.7528	50	12	0.3512	1.0
F + log.IgG3GLURPR0	5.99, 0.7872	49	12	0.3532	1.0
F + log.IgG4GLURPR0	6.13, 0.7716	50	12	0.3517	1.0
F + log.IgGGLURPR2	7.98, 0.5923	53	12	0.3518	1.0
F + log.IgG1GLURPR2	8.62, 0.6135	52	12	0.3512	1.0
F + log.IgG2GLURPR2	6.81, 0.7672	49	12	0.3514	1.0
F + log.IgG3GLURPR2	6.68, 0.7754	50	12	0.3812	1.0
F + log.IgG4GLURPR2	6.18, 0.7671	50	12	0.3909	2.0
F + log.IgGMSP1	6.60, 0.7302	51	12	0.3609	0.0
F + log.IgG1MSP1	6.03, 0.7812	50	12	0.3512	1.0
F + log.IgG2MSP1	6.53, 0.7369	50	12	0.3517	0.0
F + log.IgG3MSP1	5.78, 0.8059	49	12	0.3518	0.0
F + log.IgG4MSP1	5.81, 0.8032	49	12	0.3547	1.0
F + log.IgGMSP3	8.08, 0.5816	52	12	0.3558	0.0
F + log.IgG1MSP3	8.41, 0.6146	52	12	0.3678	1.0
F + log.IgG2MSP3	5.96, 0.7897	50	12	0.3579	2.0
F + log.IgG3MSP3	6.68, 0.7217	50	12	0.3510	1.0
F + log.IgG4MSP3	5.81, 0.8029	50	12	0.3512	2.0
F + Additive c.108C>G	7.81, 0.6743	51	12	0.3484	0.5
F + Additive c.114T>C	6.3, 0.8108	49	12	0.3501	0.5
F + Additive c.194A>G	7.42, 0.7103	50	12	0.3474	1.1
F + Additive c.233C>A	8.76, 0.5557	51	12	0.3470	1.3
F + Additive c.244A>G	8.51, 0.6066	51	12	0.3464	1.6
F + Additive c.316A>G	6.51, 0.7894	50	12	0.3483	0.6
F + Dominant c.108C>G	7.83, 0.6025	52	12	0.3455	2.1
F + Dominant c.114T>C	5.76, 0.8029	50	12	0.3458	2.0
F + Dominant c.194A>G	7.44, 0.6411	51	12	0.3467	1.5
F + Dominant c.233C>A	8.99, 0.0456	58	11	0.3166	2.4
F + Dominant c.244A>G	5.79, 0.8011	49	12	0.3491	0.1
F + Dominant c.316A>G	6.46, 0.7319	51	12	0.3476	1.0
F + Recessive c.108C>G	6.05, 0.7769	49	12	0.3454	2.2
F + Recessive c.114T>C	6.23, 0.7597	49	12	0.3487	0.3
F + Recessive c.194A>G	6.07, 0.7731	49	12	0.3480	0.7
F + Recessive c.233C>A	6.36, 0.7468	50	12	0.3500	0.4
F + Recessive c.244A>G	8.26, 0.5566	52	12	0.3470	1.3
F + Recessive c.316A>G	6.1, 0.7735	49	12	0.3479	0.8

F: Penalized maximum likelihood model (PMLE.model), LR: Likelihood Ratio test statistic; BCV: Bootstrap cross-validation; RMSE: Root Mean Squared Error; *R*^2^: Proportion of explained variation in predicted risk; AUCROC: Area Under the Receiver Operating Characteristic curve, BS: Brier score

### Evaluating the joint effect of IgGAMA1, IgG1AMA1 and dominant gene *c.*233*C*>*A*

The selection of IgGAMA1, IgG1AMA1, and dominant gene c.233C>A in the subsequent model building were based on the fact that they had a higher AUROC and R^2^, and a smaller Brier Score as previously shown in Table 3. First, the analysis involved a model which was basically the baseline model (PMLE.model) together with the three predictors (IgGAMA1, IgG1AMA1 and dominant gene *c.*233*C*>*A*) but with no shrinkage adjustment to the regression coefficients. This model was denoted as the final model with no shrinkage (FMNS). The second model was a baseline model (PMLE.model) together with the three predictors (IgGAMA1, IgG1AMA1 and dominant gene *c.*233*C*>*A*), but with shrinkage of the regression coefficient using van Houwelingen-Le Cessie heuristic estimate. This was done to improve the calibration ability of the model. This was denoted as slope corrected optimism with the van Houwelingen-Le Cessie heuristic (SCOV) model. Finally, a model was fitted and evaluated with the only IgGAMA1, IgG1AMA1 and dominant gene c.233C>A. This was denoted as the no baseline line covariate (NBC) model. The predictive performance indices of these three final models were evaluated and the results showed that the NBC model with only IgGAMA1, IgG1AMA1, and dominant gene c.233C>A predicted malaria incidence better as this model had the highest bootstrap cross-validated AUROC and *R*^2^ with a smaller Brier score as shown in Table 4.

**Table 4:** Prediction performance measures of the final selected models

Model specification	LR, p-value	BCV AUROC (97.5% CI)	BCV Brier score (%)	*R*^2^ (%)
FMNS	25.91, 0.0176	57.49(45.38–68.38)	12.10	1.00
SCOV	31.08, 0.0175	58.38(45.38–68.72)	12.00	2.00
NBC	22.41, 0.0077	61.51(48.87–70.71)	11.72	4.00

FMNS = PMLE.model + logIgGAMA1 + logIgG1AMA1 + dominant c.233 with 5 knots restricted cubic spline, Model SCOV = Slope Corrected final model with van Houwelingen-Le Cessie heuristic estimate

NBC = No baseline variable included: only dominant c.233 + logIgGAMA1 + logIgG1AMA1

LR, BCV, RMSE, *R*^2^, AUROC, BS, represent the Likelihood Ratio test statistic, bootstrap cross-validation, Root Mean Squared Error, the proportion of explained variation in predicted risk and Area Under the Receiver Operating Characteristic curve, Brier score, respectively

### Assessing the prediction performance of the three models using calibration plots

**Fig. 2:**
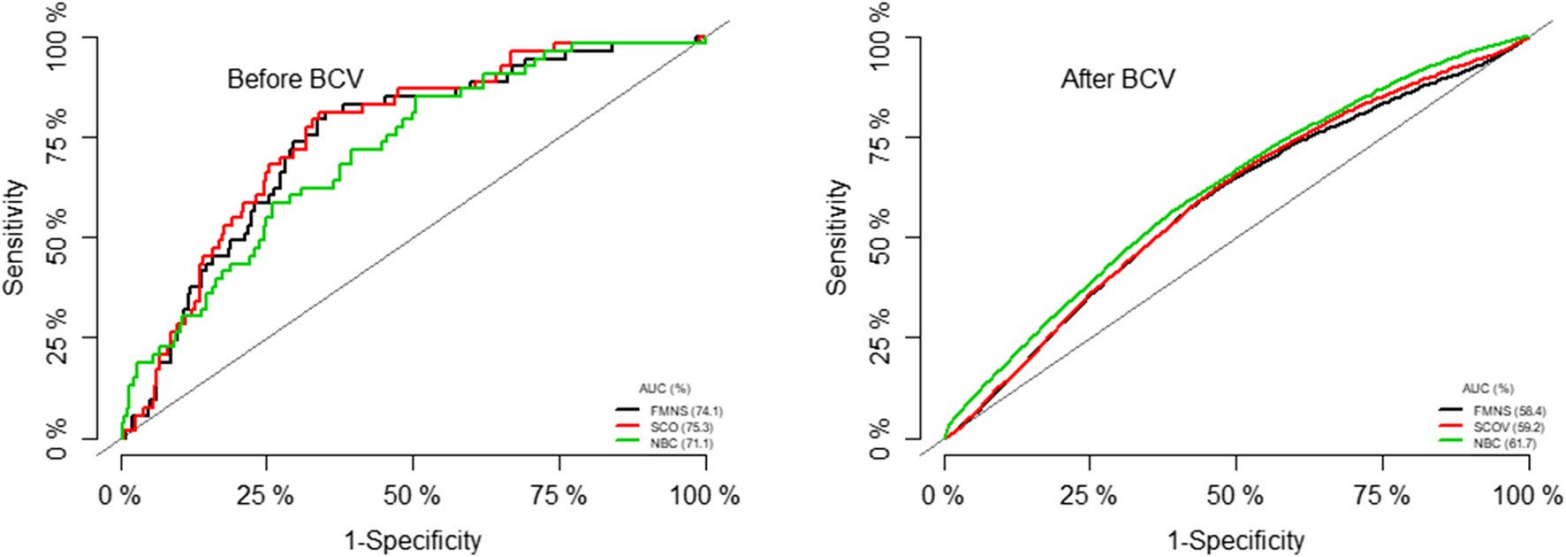
Discrimination and calibration ability of the three final models. FMNS = Final model with no shrinkage, *SCOV* Final model with slope corrected optimism using Van Howelligen estimator, *NBV* Model with no baseline variables (only IgGAMA1, IgG1AMA1, and dominant gene c.233C>A); *BCV* Bootstrap cross-validation

The prediction performance of these models was explored by examining calibration plots, that is the plots of sensitivity versus (1-specificity) (AUROC). There was no significant difference in their respective areas under the curve before internal validation, although the model with penalty corrected parameter estimates (SCOV model) had a higher AUROC (75.3%). After internally validating these models, it was observed that the AUROC of the model with only IgGAMA1, IgG1AMA1, and dominant gene c.233C>A (NBC model) performed better than the other two models (AUROC = 61.5%) (Fig. 2).

**Fig. 3:**
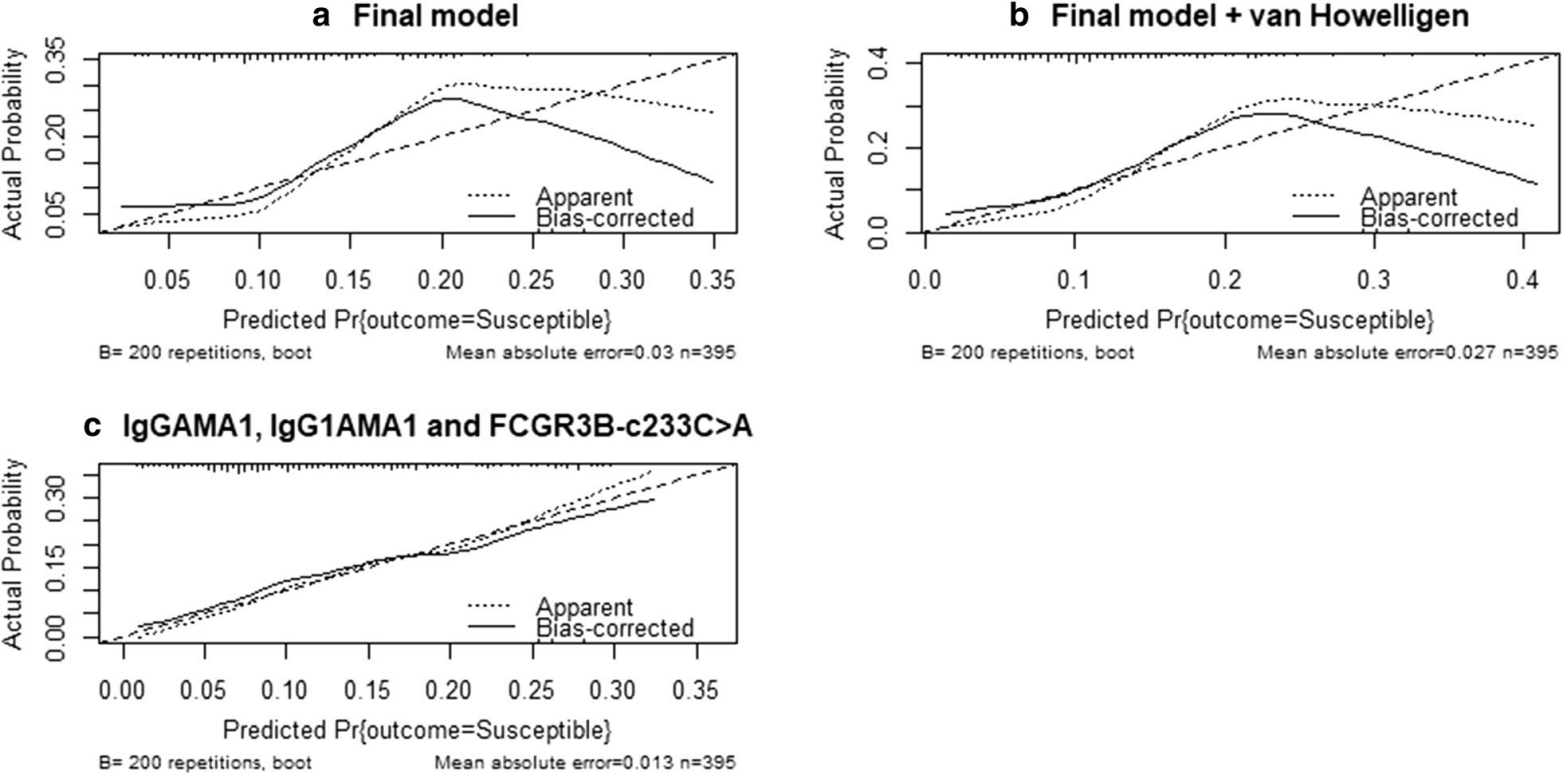
Calibration plots comparing the three final selected models

The calibration plots in Fig. 3 shows that the same model was well calibrated as it appears to be closer to the 45° line compared to the two other models.

**Fig. 4:**
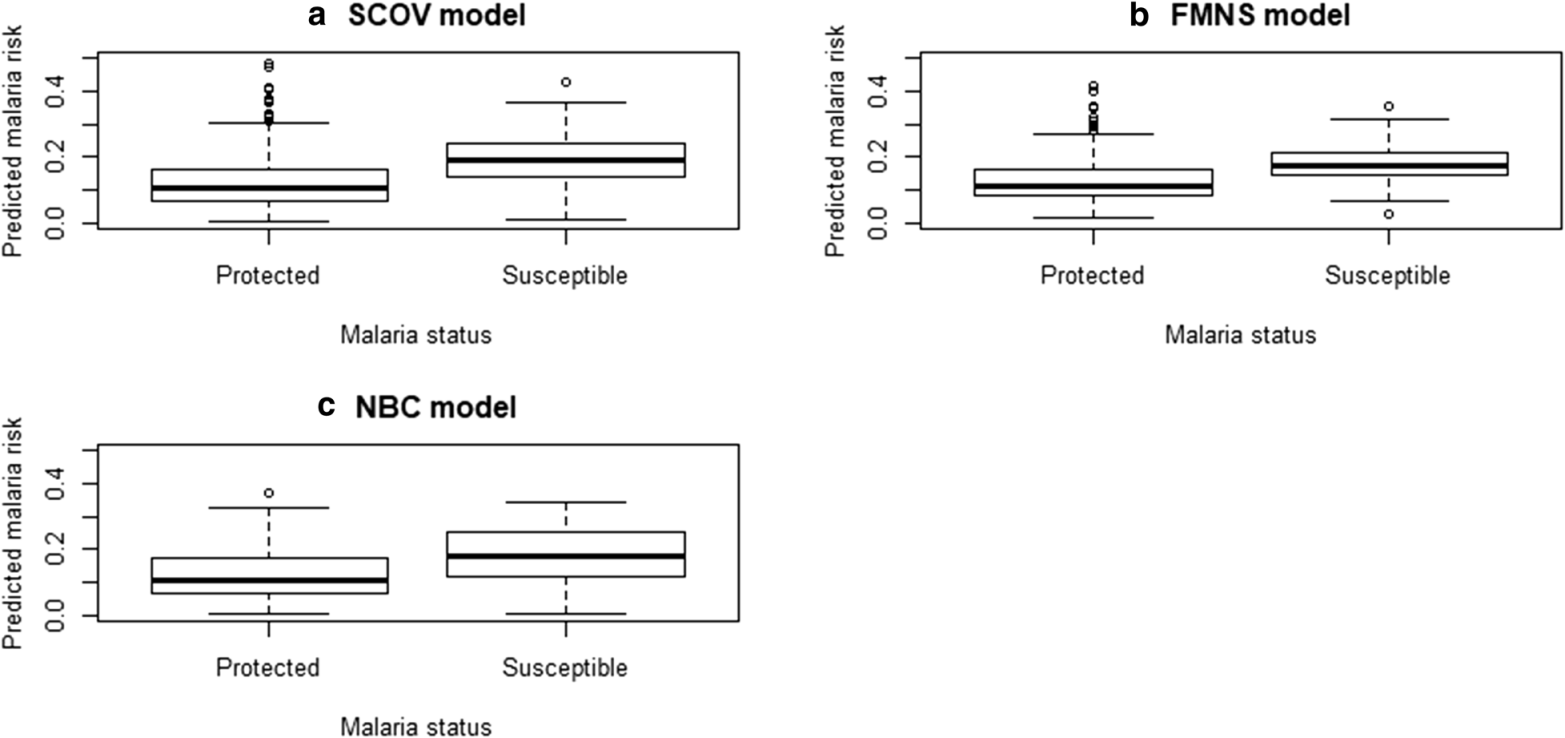
Evaluating the discrimination ability of the three final selected models. Abbreviations: SCOV: slope corrected optimism model, NBC: no baseline covariate model, that is model with only IgGAMA1, IgG1AMA1, and *c.*233*C*>*A* genotype, FMNS: Parameter estimates via penalized maximum likelihood estimation

In relation to how these models assign the low and higher predicted risk of malaria using the box-whisker plots, there was not much difference between the model with slope corrected optimism and the model with only IgGAMA1, IgG1AMA1, and dominant gene *c.*233*C*>*A* although both were slightly better than the PMLE model updated with 3 more predictors (Fig. 4).

## Discussion

The study investigated the predictive performance of several malaria-specific antibodies (IgG and subclasses) and *FCGR3B* polymorphisms on the malaria risk. This was achieved by comparing a baseline malaria model with a prediction model that integrates the antibody and genetic data. Malaria prognosis of a child is an estimate of the child’s future malaria risk. The prognosis in this study is based on the child’s baseline socio-demographic characteristics, blood group, sickle cell status, the use of bed net, presence of malaria parasite in blood and malaria-specific antibodies and *FCGR3B* genotype. Most of the socio-demographic factors, malaria antigen-specific antibodies and the *FCGR3B* variant used in this study as predictors have been found to be associated with clinical malaria in various malaria seroepidemiological studies [23, 28–34]. The discrimination and calibration performance of the baseline and integrated models measured via AUROC and the Brier score were far less than the generally recommended 80% or more for AUROC and smaller Brier score (closer to zero). Although most of the antibodies did not improve the performance of the baseline model, it is worth noting that AMA1 specific antibodies and *FCGR3B*-c.233C>A under the additive and dominant models of inheritance, can discriminate children of low and higher risk of malaria. Admittedly, the improvement in AUROC and Brier score was not very substantial from the baseline model in this study but they showed signs of improving the performance of the baseline model. This finding was consistent with other studies [31, 34–38] which identified AMA1 as an important blood-stage malaria vaccine candidate.

Traditional statistical methods of estimating odds ratios, relative risk and hazard ratio’s in epidemiological studies to assess associations between antibodies, genetic polymorphisms, and clinical malaria may not adequately determine the performance of each biomarker for predicting the risk of malaria for a child [22]. An antibody or gene variant associated with protection against malaria does not necessarily imply that it can significantly discriminate between randomly chosen children with a low or high risk of malaria and consequently, will not improve model prediction performance. This may have contributed to the poor predictive performance of most of the antibodies and genotypes in this study. For classification of children into the high and low risk of malaria, statistical techniques should be used that directly address classification accuracy (e.g. Brier score, AUROC) rather than traditional regression models for assessing associations (reporting of the odds ratio, relative risk and hazard ratio’s for time to event outcomes). Studies that link antibody responses and gene polymorphisms to clinical malaria either control for age or restrict the analysis to individuals who have been exposed to *P. falciparum* [31]. In situations where they have adjusted for most of the predictors of malaria, the predictive performance of certain antibody responses was reduced and only a few remained statistically significant [31]. Other important parameters such as haemoglobin level and sickle cell status should all be controlled for in a prediction model and its predictive performance assessed [39]. The inconsistencies in malaria risk prediction model performance indices may also be due to misclassification of the outcome variable (clinical malaria). There are several different case definitions for clinical malaria based on different parasitaemia threshold values and what would have been recorded as a case (malaria) in a particular study may be recorded as control (no malaria) in a different study and vice versa. The number of events (malaria cases) studied is important for prognostic research [41] because there is the risk of overestimating the predictive performance of the model when the number of predictors is much larger than the number of outcome events (malaria). Besides, different prognostic studies have suggested that for each candidate predictor (antibodies, host genes, age, sickle cell, haemoglobin, parasite density) studied, at least 10 events (malaria cases) are required [40–43] but these numbers could be lower in certain circumstances [44]. If for instance the number of cases studied is 200 based on fever with a threshold of 2500 parasites per microlitre of blood, then there will be differences in the number of events in a different study where clinical malaria was defined as fever with any level of parasitaemia with the latter having a larger number of cases in general to improve the power of the test. So long as malaria case definitions are not clearly defined, there will be inconsistencies in results of which antibody, host genetic factor and or their interactions have higher predictive performance on the malaria risk prediction model. Thus, in practice, different modelling strategies may result in similar or very different results even if they are applied to data from the same study. This study did not quantify the predictive performance of climatic factors on the model performance because children enrolled in the study were clustered around the same endemic area with no significant difference in climatic variables. This study recommends that prospective studies may consider including climatic variables in the malaria predictive model in situations where there are climatic differences in the geographic locations of subjects being studied.

Other factors that contribute to the incidence of malaria but were not measured to assess their predictive effect may also be of interest. These include drug resistance in parasites, vector species, parasite strain and insecticide resistance in mosquitoes [32, 45–48]. The study’s inability to observe these factors may have contributed to poor model performance. The proportion of missing values on covariates was relatively high and can result in loss of statistical power, efficiency and precision of the predicted risk notwithstanding the fact that standard statistical procedures of handling missing data were adhered to. This is particularly so when advanced statistical techniques that handle missing completely at random, missing not at random and missing at random make critical but untestable assumptions about how the data went missing [39]. The proportion of missing observations was common among immunoglobulins and host genetic factors which coincidentally were key predictors of interest in the study. To accurately evaluate the effect of malaria antibodies and host genetic factors on the risk of malaria, steps should be taken to reduce to the barest minimum the proportion of missing values among candidate predictors of malaria risk as this may bias model performance metrics.

## Conclusion

This study has found that the *FCGR3B*-c.233C>A genotype and IgG against AMA1 were relatively better compared to the other antibodies and *FCGR3B* genotypes in classifying or predicting malaria risk among children. The findings support Apical Membrane Antigen 1 as an important malaria vaccine antigen while *FCGR3B*-c.233C>A under the additive and dominant models of inheritance were also identified as an important modifier of the effect of malaria protective antibodies. Furthermore, the goal of malaria etiological risk factor studies may be quite different from studies where antibodies and host genes are used in classifying a child into high or low-risk groups. Hence the statistical methods between such studies differ to a large extent. If the latter is required, then it would be appropriate to e use model discrimination and calibration indices, such as Brier score, RMSE, and AUROC.

## Data Availability

Data will be made available upon reasonable request.

## Abbreviations

AMA: Apical merozoite antigen
FCR3G: Fc gamma receptor III gene
GLURP: Glutamate rich protein
IgG: Immunoglobulin G
MSP: Merozoite surface protein
PMLE: Penalized maximum likelihood estimate
RMSE: Root mean squared error
AUROC: Area under the receiver operating characteristic curve
IRB: Institutional review board
CI: Confidence interval
AIA2: Afro immuno assay 2
FMNS: Final model with no shrinkage
SCOV: Slope corrected optimism model
NBC: No baseline covariate model,
NBV: Model with no baseline variables
BCV: Bootstrap cross-validation

## References

[1] WHO (2016). World malaria report 2015.

[2] Hviid L (1998). Clinical disease, immunity and protection against *Plasmodium falciparum* malaria in populations living in endemic areas. Expert Rev Mol Med.

[3] Gonçalves BP, Kapulu MC, Sawa P, Guelbéogo WM, Tiono AB, Grignard L (2017). Examining the human infectious reservoir for *Plasmodium falciparum* malaria in areas of differing transmission intensity. Nat Commun..

[4] Adu B, Jepsen MP, Gerds TA, Kyei-Baafour E, Christiansen M, Dodoo D (2014). Fc gamma receptor 3B (FCGR3B-c. 233C>A-rs5030738) polymorphism modifies the protective effect of malaria specific antibodies in Ghanaian children. J Infect Dis..

[5] Salmon JE, Millard SS, Brogle NL, Kimberly RP (1995). Fc gamma receptor IIIb enhances Fc gamma receptor IIa function in an oxidant-dependent and allele-sensitive manner. J Clin Invest..

[6] Bouharoun-Tayoun H, Oeuvray C, Lunel F, Druilhe P (1995). Mechanisms underlying the monocyte-mediated antibody-dependent killing of *Plasmodium falciparum* asexual blood stages. J Exp Med..

[7] Baker SG, Kramer BS, Srivastava S (2002). Markers for early detection of cancer: statistical guidelines for nested case-control studies. BMC Med Res Methodol.

[8] Boyko EJ, Alderman BW (1990). The use of risk factors in medical diagnosis: opportunities and cautions. J Clin Epidemiol.

[9] Kattan MW (2003). Judging new markers by their ability to improve predictive accuracy. J Natl Cancer Inst.

[10] Emir B, Wieand S, Su JQ, Cha S (1998). Analysis of repeated markers used to predict progression of cancer. Stat Med.

[11] Gail MH, Costantino JP (2001). Validating and improving models for projecting the absolute risk of breast cancer. J Natl Cancer Inst.

[12] Rockhill B, Spiegelman D, Byrne C, Hunter DJ, Colditz GA (2001). Validation of the Gail et al. model of breast cancer risk prediction and implications for chemoprevention. J Natl Cancer Inst..

[13] Park Y, Freedman AN, Gail MH, Pee D, Hollenbeck A, Schatzkin A (2009). Validation of a colorectal cancer risk prediction model among white patients age 50 years and older. J Clin Oncol.

[14] Moons KGM, Kengne AP, Grobbee DE, Royston P, Vergouwe Y, Altman DG (2012). Risk prediction models: II. External validation, model updating, and impact assessment. Heart..

[15] Ridker PM, Paynter NP, Rifai N, Gaziano JM, Cook NR (2008). C-reactive protein and parental history improve global cardiovascular risk prediction: the Reynolds Risk Score for men. Circulation.

[16] Wilson PWF, D’Agostino RB, Levy D, Belanger AM, Silbershatz H, Kannel WB (1998). Prediction of coronary heart disease using risk factor categories. Circulation.

[17] Etzel CJ, Kachroo S, Liu M, D’Amelio A, Dong Q, Cote ML (2008). Development and validation of a lung cancer risk prediction model for African-Americans. Cancer Prev Res..

[18] Folsom AR, Chambless LE, Ballantyne CM, Coresh J, Heiss G, Wu KK (2006). An assessment of incremental coronary risk prediction using C-reactive protein and other novel risk markers: the atherosclerosis risk in communities study. Arch Intern Med.

[19] Tice JA, Cummings SR, Ziv E, Kerlikowske K (2005). Mammographic breast density and the Gail model for breast cancer risk prediction in a screening population. Breast Cancer Res Treat.

[20] Hubert HB, Feinleib M, McNamara PM, Castelli WP (1983). Obesity as an independent risk factor for cardiovascular disease: a 26-year follow-up of participants in the Framingham Heart Study. Circulation.

[21] Benjamin EJ, Levy D, Vaziri SM, D’Agostino RB, Belanger AJ, Wolf PA (1994). Independent risk factors for atrial fibrillation in a population-based cohort: the Framingham Heart Study. JAMA.

[22] Pepe MS, Janes H, Longton G, Leisenring W, Newcomb P (2004). Limitations of the odds ratio in gauging the performance of a diagnostic, prognostic, or screening marker. Am J Epidemiol.

[23] Adu B, Jepsen MPG, Gerds TA, Kyei-Baafour E, Christiansen M, Dodoo D (2013). Fc gamma receptor 3B (FCGR3B-c. 233C>A-rs5030738) polymorphism modifies the protective effect of malaria specific antibodies in Ghanaian children. J Infect Dis..

[24] Gerds TA, Cai T, Schumacher M (2008). The performance of risk prediction models. Biom J..

[25] Harrell Jr FE, Dupont MC, Hmisc D. The design package. R Packag version. 2007;2.

[26] Goeman JJ (2010). L1 penalized estimation in the Cox proportional hazards model. Biom J..

[27] Verweij PJ, Van Houwelingen HC (1994). Penalized likelihood in Cox regression. Stat Med.

[28] Mockenhaupt FP, Ehrhardt S, Cramer JP, Otchwemah RN, Anemana SD, Goltz K (2004). Hemoglobin C and resistance to severe malaria in Ghanaian children. J Infect Dis.

[29] Williams TN, Mwangi TW, Wambua S, Alexander ND, Kortok M, Snow RW (2005). Sickle cell trait and the risk of *Plasmodium falciparum* malaria and other childhood diseases. J Infect Dis.

[30] Aidoo M, Terlouw DJ, Kolczak MS, McElroy PD, ter Kuile FO, Kariuki S (2002). Protective effects of the sickle cell gene against malaria morbidity and mortality. Lancet.

[31] Greenhouse B, Ho B, Hubbard A, Njama-Meya D, Narum DL, Lanar DE (2011). Antibodies to *Plasmodium falciparum* antigens predict a higher risk of malaria but protection from symptoms once parasitemic. J Infect Dis.

[32] Tiendrebeogo RW, Adu B, Singh SK, Dziegiel MH, Nébié I, Sirima SB, et al. Antibody-dependent cellular inhibition is associated with reduced risk against febrile malaria in a longitudinal cohort study involving Ghanaian children. In: Open Forum Infect Dis. Oxford University Press; 2015. 10.1093/ofid/ofv044 PMC4567085 26380342

[33] Polley SD, Conway DJ, Cavanagh DR, McBride JS, Lowe BS, Williams TN (2006). High levels of serum antibodies to merozoite surface protein 2 of *Plasmodium falciparum* are associated with reduced risk of clinical malaria in coastal Kenya. Vaccine..

[34] Mitchell GH, Thomas AW, Margos G, Dluzewski AR, Bannister LH (2004). Apical membrane antigen 1, a major malaria vaccine candidate, mediates the close attachment of invasive merozoites to host red blood cells. Infect Immun..

[35] Remarque EJ, Faber BW, Kocken CHM, Thomas AW (2008). Apical membrane antigen 1: a malaria vaccine candidate in review. Trends Parasitol..

[36] Malkin EM, Diemert DJ, McArthur JH, Perreault JR, Miles AP, Giersing BK (2005). Phase 1 clinical trial of apical membrane antigen 1: an asexual blood-stage vaccine for *Plasmodium falciparum* malaria. Infect Immun.

[37] Polley SD, Mwangi T, Kocken CHM, Thomas AW, Dutta S, Lanar DE (2004). Human antibodies to recombinant protein constructs of *Plasmodium falciparum* Apical Membrane Antigen 1 (AMA1) and their associations with protection from malaria. Vaccine..

[38] Stowers AW, Kennedy MC, Keegan BP, Saul A, Long CA, Miller LH (2002). Vaccination of monkeys with recombinant *Plasmodium falciparum* apical membrane antigen 1 confers protection against blood-stage malaria. Infect Immun.

[39] Moons KGM, Royston P, Vergouwe Y, Grobbee DE, Altman DG (2009). Prognosis and prognostic research: what, why, and how?. BMJ.

[40] Harrell FE, Lee KL, Mark DB (1996). Tutorial in biostatistics multivariable prognostic models: issues in developing models, evaluating assumptions and adequacy, and measuring and reducing errors. Stat Med.

[41] Laupacis A, Sekar N, Stiell IG (1997). Clinical prediction rules. A review and suggested modifications of methodological standards. JAMA..

[42] Peduzzi P, Concato J, Feinstein AR, Holford TR (1995). Importance of events per independent variable in proportional hazards regression analysis. II. Accuracy and precision of regression estimates. J Clin Epidemiol..

[43] Peduzzi P, Concato J, Kemper E, Holford TR, Feinstein AR (1996). A simulation study of the number of events per variable in logistic regression analysis. J Clin Epidemiol.

[44] Vittinghoff E, McCulloch CE (2007). Relaxing the rule of ten events per variable in logistic and Cox regression. Am J Epidemiol.

[45] Magowan C, Wollish W, Anderson L, Leech J (1988). Cytoadherence by *Plasmodium falciparum*-infected erythrocytes is correlated with the expression of a family of variable proteins on infected erythrocytes. J Exp Med.

[46] Cherif MK, Sanou GS, Maiga B, Israelsson E, Ouédraogo AL, Bougouma EC (2012). Fc$γ$RIIa Polymorphism and anti-malaria-specific IgG and IgG subclass responses in populations differing in susceptibility to malaria in Burkina Faso. Scand J Immunol.

[47] Stanisic DI, Fowkes FJ, Koinari M, Javati S, Lin E, Kiniboro B (2015). Acquisition of antibodies against *Plasmodium falciparum* merozoites and malaria immunity in young children and the influence of age, force of infection, and magnitude of response. Infect Immun.

[48] Struthers CA, Kalbfleisch JD (1986). Misspecified proportional hazard models. Biometrika.

